# Optimism is associated with diet quality, food group consumption and snacking behavior in a general population

**DOI:** 10.1186/s12937-020-0522-7

**Published:** 2020-01-20

**Authors:** Wassila Ait-hadad, Marc Bénard, Rebecca Shankland, Emmanuelle Kesse-Guyot, Margaux Robert, Mathilde Touvier, Serge Hercberg, Camille Buscail, Sandrine Péneau

**Affiliations:** 1 Nutritional Epidemiology Research Team (EREN), Centre of Research in Epidemiology and Statistics Sorbonne Paris Cité (CRESS), Inserm U1153, Inra U1125, Cnam, Paris 13 University, 74, rue Marcel Cachin, 93017 Bobigny, France; 2 LIP/PC2S, Université de Grenoble Alpes, 38000 Grenoble, France; 3 Public Health Department, Avicenne Hospital, Bobigny, France

**Keywords:** Cross-sectional study, Diet quality, Eating behavior, Optimism, Psychology, Snacks

## Abstract

**Background:**

Dispositional optimism is a psychological trait that has been associated with positive health outcomes such as reduced risk of cardiovascular diseases. However, there is little knowledge on the relationship between optimism and dietary intake in the population. The objective of this cross-sectional study was to assess whether optimism was associated with overall diet quality, food group consumption and snacking.

**Methods:**

In 2016, 32,806 adult participants from the NutriNet-Santé study completed the Life-Orientation Test Revised (LOT-R) which assesses dispositional optimism. Overall diet quality (assessed by the mPNNS-Guideline Score) and consumption of 22 food groups were evaluated using at least three self-reported 24-h dietary records. Snacking behavior was evaluated by an ad-hoc question. Logistic and linear regressions were used to analyze the associations between optimism and these dietary behaviors, taking into account socio-demographic, lifestyle and depressive symptomatology characteristics.

**Results:**

Optimism was associated with greater overall diet quality (β (95% CI) = 0.07 (0.004–0.11), *P* < 0.0001) and higher consumption of fruit and vegetables, seafood, whole grains, fats, dairy and meat substitutes, legumes, non-salted oleaginous fruits, and negatively associated with consumption of meat and poultry, dairy products, milk-based desserts, sugar and confectionery. In addition, optimism was associated with less snacking (OR (95% CI) = 0.89 (0.84, 0.95)). In contrast, optimism was associated with higher consumption of alcoholic beverage (β (95% CI) = 5.71 (2.54–8.88), *P* = 0.0004) and appetizers (OR (95% CI) = 1.09 (1.04, 1.14)). Finally, no association was observed between optimism and energy intake.

**Conclusions:**

Optimism was associated with better overall diet quality and less snacking. It was also associated with consumption of healthy food groups as well as unhealthy food groups typically consumed in social eating occasions. These findings suggest that optimism could be taken into account in the promotion of a healthy eating behavior.

## Background

The impact of nutrition on chronic diseases, including obesity, cardiovascular diseases and cancer is utterly recognized [1–4]. Many factors influence eating behavior, including psychological ones [5]. So far, the literature has largely focused on negative psychological characteristics associated with eating behavior such as emotional eating [6, 7], cognitive restraint [8, 9] or impulsivity [10, 11]. However, more recently, research has started to focus on positive psychological traits that could have a beneficial impact on dietary behavior and nutritional status such as intuitive eating [12] or self esteem [13] . Positive characteristics generally allow the individual to easily overcome usual life stresses, perform successful work, and contribute to the social life of their community [14]. Therefore they may have positive consequences on physical health [14]. A perspective focused on building competency rather than on correcting weakness could present a major stride in health prevention [15].

One type of positive trait, called dispositional optimism is defined as the general expectation that good things, rather than bad things, will happen in the future [16]. It has been demonstrated in controlled randomized trials that optimism can be learned [17], leading to potential novel interventions to improve public health. Higher levels of dispositional optimism, assessed by validated quantitative scales, have been associated with better physical health, especially lower risk of cardiovascular diseases [18, 19], lower mortality [19, 20] and healthy aging [21]. These associations could be due to a more proactive approach to health promotion in optimistic people or better emotional responses to adversity [22]. Previous scientific literature has shown that dispositional optimism was associated with less smoking and more exercise [23]. Few studies investigated and observed an association between optimism and healthier diets. Optimism was associated with higher overall diet quality [24, 25] and greater consumption of specific healthy food groups such as fruit and vegetables [26, 27] or whole grains [28]. Although optimists are supposed to think that favorable outcomes are possible and act accordingly [29] some theorizing suggests that the positive thoughts associated with optimism might lead people to feel as if they do not need to engage in healthy behaviors [30]. Consistently, it has been observed in a cross-sectional study that dispositional optimism was associated with higher intake of alcoholic beverages [31].

Additional studies are needed since most of these studies on optimism and dietary intake have been based on specific populations such as women, older individuals or individuals with pathologies and did not consider psychological distress as potential confounders [23]. In addition, to our knowledge, no study has so far examined the association between optimism and snacking behavior. Snacking, an aspect of dietary behavior which can be defined as eating occasions apart from main meals has been associated with lower nutrient density and higher energy density compared to main meals [32], and with both healthy and unhealthy food choices [33]. In addition, greater eating frequency may contribute to an unbalanced diet that could consequently lead to overweight or obesity [34].

The aim of this study was to explore how optimism was associated with overall diet quality, energy and macronutrient intake, food group consumption and snacking behavior in a large sample of adults from the French general population, taking into account socio-demographic, lifestyle and depressive symptomatology characteristics.

## Methods

### Population

This work was conducted as part of the NutriNet-Santé study, which is a large ongoing web-based prospective cohort launched in France in May 2009 (www.etude-nutrinet-sante.fr). The rationale, design and methods of the study have been fully described elsewhere [35]. Briefly, this study was designed to investigate the relationship between nutrition and health, as well as the determinants of dietary behaviors and nutritional status. It was implemented in a general population, including Internet-using adult volunteers aged 18 or older. At inclusion, participants have to complete several self-reported web-based questionnaires to assess their diet, physical activity, anthropometric measures, lifestyle characteristics, socioeconomic conditions and health status. Participants then complete this same set of questionnaires every year after inclusion. Another set of optional questionnaires related to determinants of eating behaviors, nutritional status, and specific health-related aspects are sent each month.

The NutriNet-Santé study was conducted according to guidelines laid down in the Declaration of Helsinki. All procedures were approved by the International Research Board of the French Institute for Health and Medical Research (IRB Inserm n° 0000388FWA00005831) and the Commission Nationale de l’Informatique et des Libertés (CNIL n° 908,450 and n° 909,216). Electronic informed consent was obtained from all participants. The study was registered at clinicaltrial.org (Clinical Trial no. NCT03335644).

### Data collection

#### Dispositional optimism

Dispositional optimism was measured with the French version [36] of the Life Orientation Test revised (LOT-R) [16]. The LOT-R was administered between September and December 2016 to the NutriNet-Santé cohort. The questionnaire assesses dispositional optimism, which can be defined by a generalized expectation that good things will happen [37]. The LOT-R is a 6-item self-report questionnaire with three positively worded statements (e.g. ‘In uncertain times, I usually expect the best’) and three negatively worded statements (e.g. ‘If something can go wrong for me, it will’). Each item scored on a 5-point Likert scale ranging from 0 (strongly disagree) to 4 (strongly agree). After reversing the scoring for the negatively worded items, item scores were summed up and divided by the number of item leading to an overall optimism score ranging from 0 to 4 with higher scores representing greater optimism. We considered the LOT-R as an unidimensional scale following recommendations [38, 39]. In our population, the LOT-R displayed good internal consistency (Cronbach’s α = 0.84).

#### Assessment of food group consumption and diet quality

In the NutriNet-Santé study, all participants are invited to complete 24-h dietary records at baseline and every 6 months thereafter. The three self-administrated non-consecutive validated 24 h dietary records were randomly distributed between week and weekend days (2 weekdays and 1 weekend day). In the present study, we selected participants who completed at least three dietary records between the 2 years preceding and the 18 months following the completion of the LOT-R questionnaire, therefore between 2014 and 2018. The dietary record is completed by using an interactive interface allowing the selection of > 3300 food or beverage items and is designed for self-administration on the Internet [40]. Participants report all foods and beverages consumed at breakfast, lunch, dinner, and other eating occasions. They estimate the amounts eaten using standard measurements or using validated photographs [41]. Participants can choose among 7 portion sizes for most food products: 3 main portion sizes plus 2 intermediate and 2 extreme sizes. Nutrient intakes are estimated by using the published NutriNet-Santé food composition table [42]. Mean daily food intake (in grams per day) is weighted for the type of day of the week (weekday or weekend). Participants with unlikely estimates of energy intake were identified as under-reporters by using the method proposed by Black [43]. Briefly, basal metabolic rate was calculated according to age, gender, weight and height using Schofield’s equations [44]. Energy requirement was based on basal metabolic rate and physical activity level. The ratio between energy intake and estimated energy requirement was calculated and individuals with ratios below Goldberg cut-off were excluded [45]. The 24 h-record used in the NutriNet-Santé cohort has been validated versus blood and urinary biomarkers [46, 47], and versus an interview by a dietitian [40]. For the present study, we defined 22 food groups: fruits and vegetables, seafood (fish and shellfish), meat and poultry, processed meat, eggs, dairy products (e.g. milk, yogurts with less than 12% of added sugar), cheese, dairy and meat substitutes (e.g. soya-based products, vegetarian steaks), milk-based desserts (e.g. flan, cream desserts), starchy food, whole grains, legumes, fats (oil, butter, and margarine), sugary and fatty foods (e.g. cakes, chocolate, ice cream, pancakes), sugar and confectionery (e.g. honey, jelly, sugar, candy), fast food (e.g. pizzas, hamburgers, sandwiches, hot dogs), appetizers which included salted non oleaginous appetizers (e.g. crisps, salted biscuits) and salted oleaginous appetizers, non-salted oleaginous fruits (e.g. non-salted nuts, non-salted almonds), non-alcoholic beverages (including sweetened and light non-alcoholic beverages but excluding water) and alcoholic beverages.

The overall diet quality was assessed using the modified French National Nutrition and Health Program Guideline Score (mPNNS-GS). This score is an a priori nutritional overall diet quality score reflecting adherence to the French nutritional recommendations [48]. The mPNNS-GS is based on the PNNS-GS score [48], but accounts for dietary component only, excluding the physical activity component [49]. The score includes 12 components: eight refer to food serving recommendations (fruits and vegetables; starchy foods; whole grains; dairy products; meat, eggs and fish; seafood; vegetable fat; water vs soda) and four refer to moderation of intake (added fat; salt; sweets; and alcohol). Points are deducted for overconsumption of salt, of added sugars from sweetened foods and when energy intake exceeds the energy requirement (as assessed by physical activity level and basal metabolic rate calculated using Schofield equations [44]) by more than 5%. The score has a maximum of 13.5 points, with a higher score indicating better overall diet quality.

#### Assessment of snacking

A meal pattern questionnaire was administered between April and October 2014 where snacking, defined as eating food between meals, was assessed. Participants were asked “How often do you usually snack in the daytime?” Responses were rated on a 7-point scale from “never” to “6 times or more per day, each day” and further classified into 4 frequency categories: never, < once a week, ≥ once a week (and < once a day) and ≥ once a day. A binary variable was also computed with participants who never snacked against the others.

#### Covariates

At baseline and once per year, self-administered questionnaires are used to collect data on socio-demographic, economic characteristics, and anthropometric characteristics [50]. The data closest to the date of completion of the LOT-R were used. Potential confounders of the association between optimism and dietary intake were selected based on data of the literature. They were as follow: age (years), gender, education level (primary, secondary, undergraduate, and postgraduate), occupational status (unemployed, student, self-employed and farmer, employee and manual worker, intermediate profession, managerial staff and intellectual profession, retired), and monthly income per household unit (< 1200; 1200-1799; 1800-2299; 2300-2699; 2700-3699; ≥ 3700 euros per household unit and “unwilling to answer”), smoking status (never smoker, former smoker, and current smoker), level of physical activity, BMI, and depressive symptomatology. More precisely, monthly household income was provided and estimated per consumption unit according to household composition. The number of people in the household was converted into number of consumption units (CU) according to the OECD (Organization for Economic Cooperation and Development) equivalence scale: one CU is attributed for the first adult in the household, 0.5 for other persons aged 14 or older, and 0.3 for children under 14 [51]. Physical activity was assessed using the short form of the French version of the International Physical Activity Questionnaire (IPAQ) [52]. Energy expenditure expressed in Metabolic Equivalent of Task (MET-minutes/week) was estimated and three levels of physical activity were constituted (low (< 30 min/day), moderate (30-60 min/day), and high (≥ 60 min/day)). BMI (kg/m^2^) was calculated based on self-reported weight and height [53, 54]. Participants were classified as underweight (BMI < 18.5), normal weight (18.5 ≤ BMI < 25), overweight (25 ≤ BMI < 30) and obesity (BMI ≥ 30) [55]. Depressive symptomatology was assessed with the French version of the CES-D (Center for Epidemiology Studies Depression scale) [56, 57] between November 2017 and May 2018. It is a 20-item questionnaire rated on a 4-point scale with higher scores indicating higher depressive symptomatology. Participants were classified according to their level of depressive symptomatology (no vs yes) using the cutoff of 16 commonly used [56]. The CES-D had an excellent internal consistency (Cronbach’s α = 0.91) in our sample [56].

### Statistical analysis

To compare included with excluded participants, we used Student t-test, and Pearson’s chi-square test. Individual characteristics, overall diet quality, energy and macronutrient intake, food group consumption and snacking behavior were described as means and standard deviation (SD), median and interquartiles (IQRs) or percentages across the various categories. To describe the relationships between optimism and participants’ characteristics, Pearson correlations for continuous variables and general linear models for categorical variables were used. To estimate the association between optimism (independent variable), overall diet quality (dependent variable), energy and macronutrient intake (dependent variable), and food group consumption (dependent variable), we used multivariable regression analyses. Multivariable linear regressions analyses were used for dependent variables with a normal distribution. In the case of variables without a normal distribution (i.e. processed meat, eggs, dairy products and meat substitutes, milk-based desserts, legumes, fast food, appetizers, non salted oleaginous fruits), two levels were defined: no intake vs intake and binary logistic regressions were used. Finally, to measure the association between optimism and snacking, we used binary (no vs yes) and multinomial logistic regressions (4 frequency categories: never, < once a week, ≥ once a week (and < once a day) and ≥ once a day). For logistic regressions, the strength of the association was estimated by calculating adjusted odds ratios (ORs) and 95% confidence intervals (95% CI). Two models were tested. A first model was adjusted for age and gender. A second model was additionally adjusted for education level, occupational status, monthly income per household unit, energy intake (except when energy was the outcome), smoking status, physical activity, BMI and depressive symptomatology. Analyses were not stratified by gender since the interactions between optimism and gender were non-significant for most food groups. Missing data regarding confounders were handled using multiple imputations (mice method) by fully conditional specification (5 imputed datasets). All tests were 2-sided and statistical significance was set at 5%. Statistical analyses were performed using SAS software (SAS Institute Inc., version 9.4).

## Results

### Characteristics of the sample

A total of 32,806 participants of NutriNet-Santé study completed the optional LOT-R questionnaire from the 159,351 subjects who received it. A total of 78 participants were excluded because they presented an acquiescence bias (agreeing to all questions without consideration of reversed items). From the 32,728 remaining participants 19,335 had completed at least 3 valid 24-h dietary records (excluding under-reporters), 17,849 had available data to calculate the mPNNS-GS and 28,948 had completed the snacking behavior assessment (see Additional file 1: Figure S1). Therefore, each analysis was carried on a different subsample to make better use of available data. Compared with excluded participants (those who completed the LOT-R but less than three 24-h dietary records), included participants had a higher level of optimism (LOT-R) (2.54 ± 0.64 vs 2.48 ± 0.66), were older (56.1 ± 13.8-year-old vs 53.2 ± 14.0), had a higher proportion of men (27.5% vs 24.5%) and of individuals with university education (69.9% vs 65.4) (all *P* < 0.0001).

Table 1 describes individual characteristics of participants and the association with optimism. On average, optimism was higher in men, in individuals with a higher level of education, in self-employed or farmer, in managerial staff or intellectual professions and in individuals with higher monthly household income. In addition, optimism was higher in individuals with a normal range BMI or overweight, in individuals with higher physical activity level, in smokers (former or current) and in individuals with no depressive symptomatology. No association with age was observed. Table 2 shows the descriptive characteristics of overall diet quality, energy, macronutrient intake, food group consumption and snacking. Around three quarters of the participants declared snacking practices, among which a majority snacked more than once a week (and less than once a day).

**Table 1:** Individual characteristics of 19,335 participants (NutriNet-Santé study, 2016)

	% or Mean ± SD	LOT-R	*P*^1^
All		2.54 ± 0.64^a^	
Gender, %			< 0.0001
Male	27.48	2.58 ± 0.60	
Female	72.52	2.53 ± 0.65	
Age (years)	56.09 ± 13.76	0.0089 (−0.0052, 0.023)^b^	0.21
Education level, %			< 0.0001
Primary	2.12	2.46 ± 0.55	
Secondary	27.96	2.48 ± 0.62	
Undergraduate	31.64	2.55 ± 0.64	
Postgraduate	38.23	2.59 ± 0.65	
Missing data	0.05		
Occupational status, %			< 0.0001
Unemployed	8.78	2.47 ± 0.72	
Student	1.17	2.38 ± 0.78	
Self-employed, farmer	1.49	2.68 ± 0.65	
Employee, manual worker	9.94	2.45 ± 0.69	
Intermediate professions	12.89	2.55 ± 0.65	
Managerial staff, intellectual profession	20.17	2.62 ± 0.64	
Retired	45.57	2.54 ± 0.60	
Monthly income per household unit, %^c^			< 0.0001
< 1200€	7.59	2.45 ± 0.74	
1200–1799€	18.71	2.50 ± 0.65	
1800–2299€	15.34	2.50 ± 0.65	
2300–2699€	11.25	2.56 ± 0.62	
2700–3699€	19.84	2.60 ± 0.60	
> 3700€	15.72	2.64 ± 0.62	
Unwilling to answer	11.27	2.47 ± 0.62	
Missing data	0.28		
BMI (kg/m^2^), %			< 0.0001
< 18.5	5.06	2.44 ± 0.71	
18.5–24.99	65.38	2.57 ± 0.63	
25–29.99	22.27	2.53 ± 0.62	
≥ 30	7.27	2.43 ± 0.70	
Missing data	0.02		
Physical activity, %			< 0.0001
Low	20.92	2.47 ± 0.66	
Moderate	40.16	2.54 ± 0.64	
High	38.89	2.58 ± 0.62	
Missing data	0.04		
Smoking status, %			0.019
Never-smoker	51.44	2.53 ± 0.65	
Former smoker	40.40	2.56 ± 0.62	
Current smoker	8.16	2.55 ± 0.67	
Depressive symptomatology (CES-D^d^), %			< 0.0001
Yes (≥16)	15.19	2.03 ± 0.65	
No (< 16)	68.08	2.66 ± 0.58	
Missing data	16.73		

LOT-R, Life Orientation Test-Revised, score ranges from 0 to 4, the highest score corresponds to highest optimism

^1^p-value based on linear regressions for categorical variables or Fisher’s z transformation for continuous variables

^a^Mean ± SD, all such values

^b^Pearson correlations (95% CI), all such values

^c^Monthly income represents the household income per month calculated by consumption unit (CU). The number of people of the household was converted into a number of CU according to a weighting system: one CU is attributed for the first adult in the household, 0.5 for other persons aged 14 or older and 0.3 for children under 14

^d^CES-D, Center for Epidemiology Studies Depression scale, score ranges from 0 to 60 the highest score corresponds to highest depressive symptomatology

**Table 2:** Descriptive characteristics of diet quality, energy and macronutrient intake, food group consumption, and snacking behavior (NutriNet-Santé study, 2016)

	Mean ± SD or Median [IQRs] or %
Diet quality (*N* = 17,849)	
mPNNS-GS^a^	7.66 ± 1.51^b^
Energy and macronutrients (*N* = 19,335)	
Energy intake, kcal/d	1916.52 ± 427.08
Energy intake without alcohol, kcal/ d	1855.86 ± 409.57
Protein, %	16.03 ± 2.90
Carbohydrate, %	41.25 ± 6.59
Lipid, %	39.42 ± 5.69
Food group consumption (N = 19,335)	
Fruit and vegetables, g/d	485.14 ± 234.77
Seafood, g/d	36.40 ± 33.33
Meat and poultry, g/d	68.68 ± 44.26
Processed meat, g/d	12.14 [1.86, 25.76]^c^
Eggs, g/d	8.93 [0.00, 21.11]
Dairy products, g/d	140.01 ± 136.82
Cheese, g/d	35.97 ± 26.67
Dairy and meat substitutes, g/d	0.00 [0.00, 0.00]
Milk-based desserts, g/d	16.43 [0.00, 44.64]
Starchy foods, g/d	219.01 ± 90.31
Whole grains, g/d	40.90 ± 47.74
Legumes, g/d	0.00 [0.00, 17.86]
Fats, g/d	21.41 ± 13.95
Sugary and fatty foods, g/d	72.64 ± 54.34
Sugar and confectionery, g/d	30.31 ± 28.68
Fast food, g/d	20.09 [0.00, 47.93]
Appetizers, g/d	1.79 [0.00, 7.14]
Salted non oleaginous appetizers, g/d	0.00 [0.00, 4.76]
Salted oleaginous appetizers, g/d	0.00 [0.00, 1.43]
Non-salted oleaginous fruits, g/d	1.12 [0.00, 6.24]
Non-alcoholic beverages, g/d	552.42 ± 352.37
Alcoholic beverages, g/d	104.56 ± 141.76
Snacking behavior (*N* = 28,948)	
Overall snacking, %	
No	15.46
Yes	84.54
Snacking frequencies, %	
Never	15.46
< once a week	24.42
≥ once a week (and < once a day)	42.03
≥ once a day	18.10

^a^mPNNS-GS, modified French National Nutrition and Health Program Guideline Score

^b^Mean ± SD, all such values

^c^Median [IQRs], all such values

#### Association between optimism, diet quality, and intakes of energy, macronutrients and food groups

Tables 3 and 4 present the association between optimism and dietary intake. A higher score of optimism was associated with a greater overall diet quality (mPNNS-GS), higher intake of lipids (% of energy) and lower intake of carbohydrates and proteins (% of energy). No association with energy (nor energy without alcohol) was observed. Regarding food groups, participants with higher optimism reported higher consumption of fruits and vegetables, seafood, whole grains, fats, dairy and meat substitutes, legumes, appetizers (including salted non oleaginous appetizers and salted oleaginous appetizers), non-salted oleaginous fruits and alcoholic beverage. They also had lower intakes of meat and poultry, dairy products, milk-based desserts and sugar and confectionery.

**Table 3:** Linear regression analyses showing association between optimism, diet quality, energy and macronutrient intake and food group consumption (NutriNet-Santé study, 2016)

Outcomes	Βeta-coefficient (95% CI)		
	LOT-R		*P*^3^
	Model 1^1^	Model 2^2^	
Diet quality (N = 17,849)			
mPNNS-GS^4^	0.11 (0.08, 0.15)	0.07 (0.04, 0.11)	< 0.0001
Energy and macronutrient intake (N = 19,335)			
Energy intake, kcal/d	8.02 (−0.04, 16.08)	5.66 (−3.24, 14.55)	0.21
Energy intake without alcohol, kcal/ d	4.19 (− 3.73, 12.12)	2.37 (−6.33, 11.08)	0.59
Protein, %	−0.19 (0.25, − 0.13)	−0.16 (− 0.23, − 0.09)	< 0.0001
Carbohydrate, %	−0.34 (− 0.48, 0.19)	−0.32 (− 0.47, − 0.16)	< 0.0001
Lipid, %	0.33 (0.20, 0.45)	0.31 (0.17, 0.44)	< 0.0001
Food group consumption (*N* = 19,335)			
Fruit and vegetables, g/d	12.89 (7.86, 17.92)	7.76 (2.42, 13.10)	0.0044
Seafood, g/d	1.49 (0.77, 2.21)	1.09 (0.31, 1.88)	0.0064
Meat and poultry, g/d	−2.20 (−3.15, −1.25)	−2.07 (− 3.07, −1.07)	< 0.0001
Dairy products, g/d	−8.35 (− 11.36, −5.34)	−8.84 (− 12.12, −5.58)	< 0.0001
Cheese, g/d	0.33 (−0.25, 0.91)	0.10 (−0.51, 0.71)	0.74
Starchy foods, g/d	−0.63 (− 2.48, 1.22)	− 0.94 (− 2.76, 0.89)	0.31
Whole grains, g/d	1.96 (0.92, 3.01)	1.63 (0.49, 2.76)	0.005
Fats, g/d	0.30 (−0.005, 0.60)	0.37 (0.05, 0.69)	0.023
Sugary and fatty foods, g/d	0.63 (−0.40, 1.65)	−0.52 (−1.70, 0.67)	0.39
Sugar and confectionery, g/d	−0,52 (−1.14, 0.11)	− 0.85 (− 1.51, − 0.20)	0.011
Non-alcoholic beverages, g/d	16.46 (8.77, 24.15)	5.48 (−2.95, 13.92)	0.21
Alcoholic beverages, g/d	6.87 (3.91, 9.82)	5.71 (2.54, 8.88)	0.0004

^1^Model adjusted for age and gender

^2^Model adjusted for age, gender, education level, occupational status, monthly income per household unit, energy intake (except for the model where energy intake was the outcome) BMI and depressive symptomatology

^3^P value based on linear regressions adjusted for age, gender, education level, occupational status, monthly income per household unit, energy intake (except for the model where energy intake was the outcome) BMI and depressive symptomatology, with optimism as a continuous independent variable

^4^mPNNS-GS, modified French National Nutrition and Health Program Guideline Score

**Table 4:** Logistic regression analyses showing association between optimism and food group consumption (NutriNet-Santé study, 2016)

Outcomes	LOT-R		
	Odds ratio (95% CI)		*P*^3^
	Model 1^1^	Model 2^2^	
Food group consumption (*N* = 19,335)			
Processed meat			
No intake	Ref^4^	Ref	
Intake	1.007 (0.96, 1.06)	0.96 (0.91, 1.02)	0.18
Eggs			
No intake	Ref	Ref	
Intake	1.05 (1.00, 1.10)	1.01 (0.96, 1.07)	0.65
Dairy and meat substitutes			
No intake	Ref	Ref	
Intake	1.15 (1.09, 1.21)	1.17 (1.11, 1.24)	< 0.0001
Milk-based desserts			
No intake	Ref	Ref	
Intake	0.94 (0.90, 0.98)	0.94 (0.89, 0.99)	0.011
Legumes			
No intake	Ref	Ref	
Intake	1.09 (1.04, 1.14)	1.07 (1.02, 1.13)	0.005
Fast food			
No intake	Ref	Ref	
Intake	1.02 (0.98, 1.08)	0.99 (0.94, 1.05)	0.80
Appetizers			
No intake	Ref	Ref	
Intake	1.15 (1.10, 1.20)	1.09 (1.04, 1.14)	0.0007
Salted non oleaginous appetizers			
No intake	Ref	Ref	
Intake	1.11 (1.06, 1.16)	1.06 (1.01, 1.11)	0.02
Salted oleaginous appetizers			
No intake	Ref	Ref	
Intake	1.16 (1.11, 1.22)	1.11 (1.05, 1.17)	0.0001
Non salted oleaginous fruits			
No intake	Ref	Ref	
Intake	1.22 (1.17, 1.28)	1.16 (1.10, 1.22)	< 0.0001

^1^Model adjusted for age and gender

^2^Model adjusted for age, gender, education level, occupational status, monthly income per household unit, energy intake (except for the model where energy intake was the outcome) BMI and depressive symptomatology

^3^P values based on logistic regression adjusted for age, gender, education level, occupational status, monthly income per household unit, energy intake, BMI and depressive symptomatology, with optimism as a continuous independent variable

^4^Reference

#### Association between optimism and snacking behavior

Table 5 shows the association between optimism and snacking behavior. Optimism was inversely associated with overall snacking. More specifically, the association between optimism and snacking was observed for individuals who snacked at least once a week.

**Table 5:** Logistic regression analyses showing association between optimism and snacking behavior (NutriNet-Santé study, 2014)

Outcomes	LOT-R		
	Odds ratio (95% CI)		*P*^3^
	Model 1^1^	Model 2^2^	
Overall Snacking (*N* = 28,948)			
No	Ref^4^	Ref	
Yes	0.97 (0.96, 0.98)	0.89 (0.84, 0.95)	0.0003
Snacking frequencies (*N* = 28,948)			
Never	Ref	Ref	
< once a week	0.99 (0.98, 1.00)	0.98 (0.91, 1.04)	0.47
≥ once a week (and < once a day)	0.97 (0.96, 0.98)	0.88 (0.82, 0.94)	0.0002
≥ once a day	0.94 (0.93, 0.95)	0.80 (0. 74, 0.86)	< 0.0001

^1^Model adjusted for age and gender

^2^Model adjusted for age, gender, education level, occupational status, monthly income per household unit, energy intake, BMI and depressive symptomatology

^3^P values based on binary and multinomial logistic regression adjusted for age, gender, education level, occupational status, monthly income per household unit, energy intake, BMI and depressive symptomatology, with optimism as a continuous independent variable

^4^Reference

## Discussion

To our knowledge, this study is the first to assess the association between optimism and eating behavior using 24 h records and taking socio-demographic, lifestyle and depressive symptomatology characteristics into account in a very large population-based sample of adults. Our findings suggest that dispositional optimism was associated with a higher overall diet quality. Optimism was positively associated with consumption of fruits and vegetables, seafood, whole grains, fats, dairy and meat substitutes, legumes, and non-salted oleaginous fruits, and negatively associated with consumption of meat and poultry, dairy products, milk-based desserts and sugar and confectionery. However, optimism was positively associated with alcoholic beverages and appetizers. Optimism was associated with lower snacking and snacking frequency. Finally, no association with energy was found.

### Optimism, diet quality, and intakes of energy, macronutrients and food groups

We found that dispositional optimism was associated with an overall greater diet quality supporting previous data showing a positive association between optimism and alternative healthy eating index (AHEI) in postmenopausal women [24] and with a healthy diet metric of the ideal cardiovascular health based on the AHA guidelines in adults [25]. Our data indicated no association between optimism and energy, while optimism was associated with higher intake of lipids, and lower intake of proteins and carbohydrates. To our knowledge, no other study has investigated the association between optimism, energy and macronutrient intake. These results suggest that optimism could have an impact on diet quality but not on the overall energy intake. Our data showed that optimism was mainly associated with greater intake of healthy food groups including fruits and vegetables, seafood, whole grains, legumes, non-salted oleaginous fruits and lower intake of unhealthy food including milk-based desserts and sugar and confectionery. In agreement, previous data showed that higher optimism was associated with greater intake of fruits and vegetables [26, 27], and whole grains [28]. Healthier choices in optimistic individuals may be due to a more proactive approach to health promotion [22]. Optimistic individuals have been shown more likely to adopt healthier behaviors including less smoking and more exercise [23]. Optimists also show a better profile of emotional responses to adversity (less distress, more positive emotions) due to more effective coping reactions, which can lead to healthier choices [22]. Our results showing an association between optimism and dietary intake are important since it has been demonstrated in randomized trials that optimism can be learned [17], leading to potential novel interventions to improve public health. However, the reverse has also been suggested; that is, individuals who engage in healthier dietary behaviors may also as a consequence be more optimistic [58, 59].

In contrast, our findings also showed that higher optimism was associated with some unhealthy dietary items, i.e. appetizers (both oleaginous and non-oleaginous) and alcoholic beverages. In agreement, previous data showed higher intake of alcohol in elderly with higher optimism [31]. However, no association between optimism and median alcohol intake was observed in another study [27]. The large majority of meals in France is shared with others [60]. Appetizers and alcoholic beverages are typically eaten during social eating occasions and in particular in France during the so called “*apéritif*”. The *apéritif* is a moment of conviviality shared by people before the main meal and often consisting of raw vegetables, crisps, salted nuts, processed meat, cheese and quiches/pizzas [61, 62] as well as alcoholic and sweetened non-alcoholic drinks [62]. Studies have shown that optimists have greater social connections than pessimists [22, 63, 64] suggesting that they are more likely to share their meals with other people and therefore to include an *apéritif* in their meal.

### Optimism and snacking behavior

Our results showed that more optimistic individuals were less likely to snack and to snack often compared with less optimistic individuals. To our knowledge, there is no data available assessing the association between optimism and snacking behavior. Snacking has been associated with depressive symptomatology [65], perceived stress [66] and coping strategies [67]. We can hypothesize that the better coping strategies observed in more optimistic individuals [29] lead to lower psychological distress [68, 69], which can in turn decrease snacking through lower emotional eating. Indeed, emotional eating has been associated with both stress [70, 71] and snacking [7].

### Strengths and limitations

The main strength of this study was the use of at least three 24-h dietary records that allowed us to have a good representation of the participants’ usual diet. As previously shown, usual intake of population can be estimated based on at least two recalls [72]. Another important strength is the large sample size, which provided a high statistical power. The use of the Internet for data collection permitted access to a heterogeneous sample of volunteers. In addition, important socio-demographic and economic confounding factors have been taken into account. However we cannot exclude the possibility of residual confounding due to other individual or environmental factors. The Web-based version of the LOT-R questionnaire minimized missing data by using automatic controls and alerts to users. The LOT-R was validated in French and demonstrated good psychometric properties [36]. The main limitation of our study is its transversal conception which does not allow us to conclude on the causal relationship of the association. An inverse causality between dispositional optimism and dietary intake is likely to exist, as previously suggested [58, 59]. In our analysis, depressive symptom was assessed from a questionnaire collected in between 2017 and 2018 while optimism were collected in 2016. So there could be a problem of temporality. Caution is also needed when generalizing our results since the NutriNet-Santé study is a long-term nutrition-focused cohort and participants are recruited on a voluntary basis. Consequently, our subjects are likely to have high health awareness and a higher interest in nutrition compared to the French population. However, a low magnitude of differences in food intakes (apart from fruits and vegetables) was observed between the NutriNet-Santé study and a representative sample of the French population [73]. In regards to snacking measure, the nutritional quality of the snacks was not assessed in the present study, although previous data of the NutriNet-Santé cohort suggested a lower nutrient density and higher energy density of snacks compared with main meals [32]. Finally, optimism trait is regarded as a stable personality characteristic over extended periods [29]. However, it relies on the subject’s self-perception and understanding of the questionnaire.

## Conclusions

Our findings showed that dispositional optimism was associated with a higher overall diet quality, and less snacking practices. It was also associated with consumption of specific unhealthy foods and alcohol beverages. Our results therefore suggest that optimists tend to have a healthier diet overall but with larger intakes of food and beverages typically consumed at social eating occasions. Replication of these findings, particularly by longitudinal and experimental studies are needed. Since optimism can be enhanced, programs targeting optimism may provide effective strategies for helping influencing dietary behaviors toward better food choices.

## Supplementary information

**Additional file 1: Figure S1.** Participant flow chart from the NutriNet-Santé cohort study included in current analyses.

## Data Availability

Data of this study are protected under the protection of health data regulation set by the ‘Commission Nationale de l’Informatique et des Libertés (CNIL). The data are available upon request to Nathalie Pecollo (n.pecollo@eren.smbh.univ-paris13.fr) for review by the steering committee of the NutriNet-Santé study.

## Abbreviations

CES-D: Center for Epidemiologic Studies Depression scale
CNIL: Commission Nationale de l’Informatique et des Libertés
CU: Consumption Unit
IRB: Institute for Health and Medical Research
LOT-R: Life Orientation Test Revised
mPNNS-GS: modified Programme National Nutrition Santé - Guideline Score
OECD: Organization for Economic Cooperation and Development
