# Supplementary material for: Optimism is associated with diet quality, food group consumption and snacking behavior in a general population
Source: Nutr J. 2020 Jan 20;19:6. doi: 10.1186/s12937-020-0522-7 (PMC6971864; doi:10.1186/s12937-020-0522-7)
Supplement: Supplementary file 1 — Additional file 1: Figure S1. Participant flow chart from the NutriNet-Santé cohort study included in current analyses. [file 12937_2020_522_MOESM1_ESM.docx]

**Online Supporting Material**

Supplemental Figure 1: Participant flow chart from the NutriNet-Santé cohort study included in current analyses.

159,351 participants included in the NutriNet-Santé study received the LOT-R questionnaire

(September 2016)

126,545 participants who did not complete the LOT-R questionnaire were excluded

32,806 participants completed the LOT-R questionnaire

78 participants with an acquiescence bias were excluded

32,728 participants completed the LOT-R questionnaire without acquiescence bias

19,335 participants completed at least three dietary records

28,948 participants completed the snacking assessment

17,849 had available data to calculate the mPNNS-GS
